# The Expression of PHOSPHO1, nSMase2 and TNAP is Coordinately Regulated by Continuous PTH Exposure in Mineralising Osteoblast Cultures

**DOI:** 10.1007/s00223-016-0176-9

**Published:** 2016-07-21

**Authors:** D. A. Houston, K. Myers, V. E. MacRae, K. A. Staines, C. Farquharson

**Affiliations:** The Roslin Institute and R(D)SVS, University of Edinburgh, Easter Bush, Midlothian, EH25 9RG Scotland, UK

**Keywords:** Parathyroid hormone, Osteoblast, Mineralisation regulators, Hyperparathyroidism, PHOSPHO1

## Abstract

Sustained exposure to high levels of parathyroid hormone (PTH), as observed in hyperparathyroidism, is catabolic to bone. The increase in the RANKL/OPG ratio in response to continuous PTH, resulting in increased osteoclastogenesis, is well established. However, the effects of prolonged PTH exposure on key regulators of skeletal mineralisation have yet to be investigated. This study sought to examine the temporal expression of PHOSPHO1, TNAP and nSMase2 in mineralising osteoblast-like cell cultures and to investigate the effects of continuous PTH exposure on the expression of these enzymes in vitro. PHOSPHO1, nSMase2 and TNAP expression in cultured MC3T3-C14 cells significantly increased from day 0 to day 10. PTH induced a rapid downregulation of *Phospho1* and *Smpd3* gene expression in MC3T3-C14 cells and cultured hemi-calvariae. *Alpl* was differentially regulated by PTH, displaying upregulation in cultured MC3T3-C14 cells and downregulation in hemi-calvariae. PTH was also able to abolish the stimulatory effects of bone morphogenic protein 2 (BMP-2) on *Smpd3* and *Phospho1* expression. The effects of PTH on *Phospho1* expression were mimicked with the cAMP agonist forskolin and blocked by the PKA inhibitor PKI (5-24), highlighting a role for the cAMP/PKA pathway in this regulation. The potent down-regulation of *Phospho1* and *Smpd3* in osteoblasts in response to continuous PTH may provide a novel explanation for the catabolic effects on the skeleton of such an exposure. Furthermore, our findings support the hypothesis that PHOSPHO1, nSMase2 and TNAP function cooperatively in the initiation of skeletal mineralisation.

## Introduction

Phosphatases are essential regulators of skeletal mineralisation, modifying local levels of inorganic phosphate (Pi), inorganic pyrophosphate (PPi) and the phosphorylation status of osteopontin, a key regulator of extracellular matrix (ECM) mineralisation [1]. The importance of tissue-nonspecific alkaline phosphatase (TNAP) as an essential component of successful skeletal mineralisation is perhaps most striking in the condition known as hypophosphatasia (HPP). Individuals possessing hypomorphic *ALPL* (the gene encoding TNAP in humans) mutations typically present with severe rickets and osteomalacia resulting in a blockade of hydroxyapatite propagation within the ECM [2]. Despite this, chondrocyte- and osteoblast-derived matrix vesicles (MV) from both HPP patients and *Alpl* ^−/−^ mice retain the ability to initiate intra-vesicular mineralisation and contain hydroxyapatite crystals [3, 4], highlighting that TNAP is not essential for the initiation of MV-mediated ECM mineralisation.

Active within chondrocyte- and osteoblast-derived MV [5, 6] and expressed exclusively in mineralising bone, cartilage and dentin [7–9], PHOSPHO1, a member of the haloacid dehalogenase superfamily, is essential for the initiation of MV-mediated mineralisation. Small-molecule inhibition of PHOSPHO1 in MV’s derived from *Alpl* ^−/−^ mice significantly affects their ability to calcify in vitro [7]. The genetic ablation of *Phospho1* results in severely hypomineralised skeletal and dental tissues with resulting bowing of the long bones, spontaneous fractures and scoliosis [9–12]. Complete ablation of skeletal mineralisation is observed in *Phospho1* ^−/−^;*Alpl* ^−/−^ double-knockout embryos and in metatarsals cultured in the presence of both PHOSPHO1 and TNAP inhibitors [11, 13]. In vitro, PHOSPHO1 shows high phosphohydrolase activity towards phosphocholine and phosphoethanolamine, two metabolites derived from lipid metabolism [5]. More recently, hypomineralisation of skeletal and dental tissues was observed in the *fro/fro* mouse, a mouse containing a major deletion in the sphingomyelinase phosphodiesterase 3 (*Smpd3)* gene [14, 15]. *Smpd3* encodes for neutral sphingomyelinase 2 (nSMase2), which catalyses the breakdown of the membrane lipid sphingomyelin to ceramide and phosphocholine. Through their role in the generation and processing of phosphocholine, nSMase2 and PHOSPHO1, respectively, may function together to liberate Pi for skeletal mineralisation. In spite of our knowledge of the skeletal pathophysiology in the absence of *Phospho1*, little is known about the regulation of *Phospho1* expression.

A recent RNA-seq analysis by St John et al. [16] revealed that *Phospho1* and *Smpd3* expression are differentially regulated by parathyroid hormone (PTH) treatment of IDG-SW3 cells (an osteocyte cell line). This study extends a growing body of evidence revealing that PTH can regulate a plethora of genes involved in osteoblast function [17]. Indeed, a microarray study of PTH-treated UMR-106-01 cells, a rat osteosarcoma cell line, revealed >100 differentially regulated genes, with the gene profile closely mimicking the gene expression pattern observed during osteoblast differentiation [18]. The transcription and posttranslational modification (phosphorylation) of Cbfa1, the transcription factor and master regulator of osteoblast differentiation are also strongly enhanced by PTH [19]. Alterations in osteoblast gene expression by PTH may underscore PTH ability to elicit both anabolic and catabolic effects on the skeleton depending on the duration of the exposure. For example, daily injections of low-dose PTH are anabolic to the skeleton, whereas continuous exposure to PTH, the likes observed in hyperparathyroidism, results in bone loss and increased porosity especially in the cortical compartment [20]. Indeed, despite an overall increase in bone remodelling [21], the enhancement of receptor activator of nuclear factor-κB ligand (RANKL) expression and thus osteoclastogenesis, and the suppression of the RANKL decoy receptor, osteoprotegerin [22], ensure that any increases in bone formation are outweighed by a prevailing bone resorption response. Furthermore, the bone formation response that occurs is predominated by osteoid production rather than a true mineralised bone matrix [23]. The reduction in bone mineral density associated with hyperparathyroidism [24] may be a direct effect of PTH on key regulators of mineralisation. This study, therefore, sought to examine the effect of a short-term continuous PTH exposure on PHOSPHO1, TNAP and nSMase2 expression in osteoblast-like cells and calvariae explants as well as to examine the PTH signal transduction pathways involved.

## Results

### Temporal Gene and Protein Expression and Matrix Mineralisation in MC3T3 Cells

**Fig. 1:**
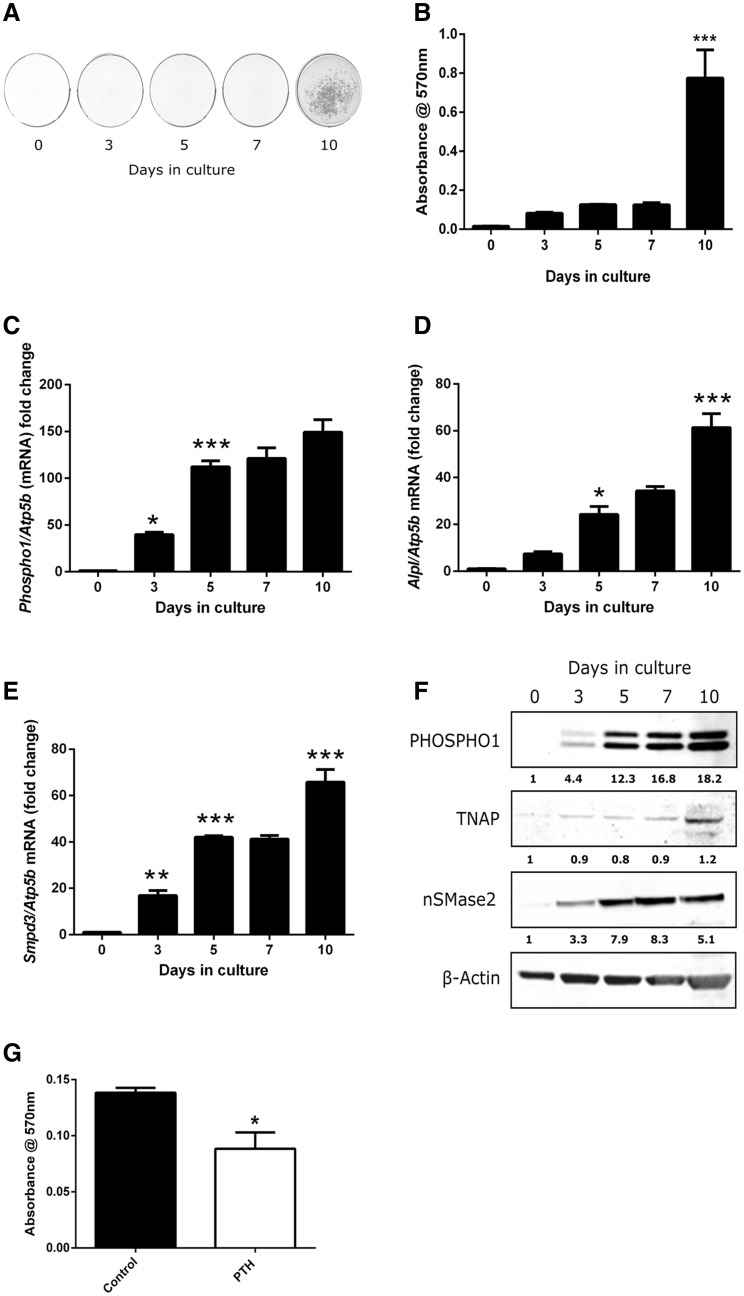
Extracellular matrix mineralisation and temporal gene expression in MC3T3-C14 cells. **a** Alizarin red staining and **b** relative quantification of cetylpyridinium chloride leached bound dye. RT-qPCR analysis of **c** *Phospho1* **d** *Alpl* and **e** *Smpd3* mRNA expression in mineralising MC3T3-C14 cells over a 10-day culture period. **f** Western blotting analysis of PHOSPHO1, TNAP and nSMase2 in MC3T3-C14 cells over a 10-day culture period. The fold change in fluorescence intensity against cultures at day 0 (normalised against beta-actin) is shown below each protein of interest. **g** Assessment of matrix mineralisation in MC3T3-C14 cell cultures continuously exposed to PTH for 10 days. Quantification of, cetylpyridinium chloride leached, bound Alizarin red staining. *N* = 3; *P < 0.05, **P < 0.01; ***P < 0.001 in comparison with the previous time point in culture

MC3T3-C14 cells cultured in the presence of ascorbic acid and calcium chloride mineralised their ECM by day 10. Quantification of leached Alizarin red confirmed a significant increase in mineral content on day 10 compared with day 7 of culture (0.775 ± 0.145 vs. 0.125 ± 0.01 relative absorbance units, *P* < 0.001; Fig. 1a, b). *Phospho1* mRNA expression markedly increased in a temporal manner and by day 10 in culture mRNA levels were ~ 150-fold higher (*P* < 0.001) compared to day 0 (Fig. 1c). A similar temporal increase in *Alpl* and *Smpd3* was also noted and by day 10 in culture where the expression of both was increased by >60-fold (*P* < 0.001) compared to day 0 (Fig. 1d, e). The changes in gene expression were similarly observed at the protein level by western blotting (Fig. 1f). The expression of both PHOSPHO1 protein bands, which are considered to be a result of alternative start sites, was increased similarly with time in culture [8].

### The Effects of Continuous PTH on Matrix Mineralisation in MC3T3 Cells

The addition of 50 nM PTH every 48 h to MC3T3-C14 cell cultures for 10 days caused a reduction in the extent of matrix mineralisation compared to control cultures as assessed by the quantitative alizarin red assay (*P* < 0.05; Fig. 1g).

### The Effects of Continuous PTH on Gene and Protein Expression in MC3T3 Cells

**Fig. 2:**
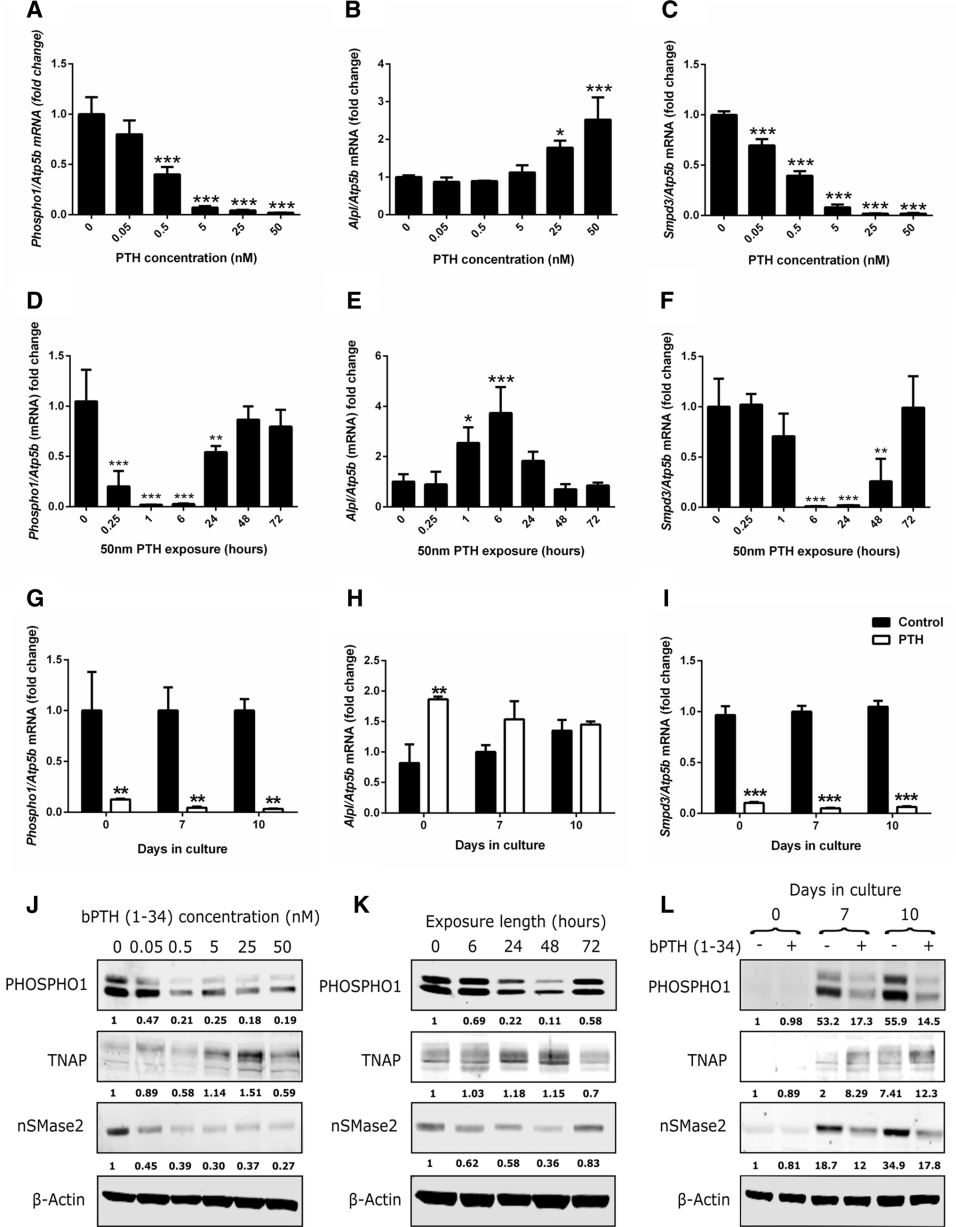
Regulation of key mineralisation genes by PTH in MC3T3-C14 cells. RT-qPCR analysis of **a** *Phospho1*, **b** *Alpl* and **c** *Smpd3* mRNA expression in response to a 24-h exposure of various doses of bPTH (1–34) on day 6 of culture. RT-qPCR analysis of **d** *Phospho1*, **e** *Alpl* and **f** *Smpd3* mRNA expression in response to various exposure times of bPTH (1–34) (50 nM). The timing of PTH addition was adjusted to ensure all experiments finished on day 7 of culture. RT-qPCR analysis of **g** *Phospho1*, **h** *Alpl* and **i** *Smpd3* mRNA expression in response to 24-h exposure PTH (50 nM) in MC3T3-C14 cells at different stages of differentiation (confluency (day 0), days 7 and day 10 post-confluency). **j** Western blotting analysis of PHOSPHO1, TNAP, nSMase2 in response to a 24-h exposure of various doses of PTH. The fold change in fluorescence intensity against control treated cultures (normalised against beta-actin) is shown below each protein of interest. **k** Western blotting analysis of PHOSPHO1, TNAP, nSmase2 in response to various exposure times of PTH (50 nM). The fold change in fluorescence intensity against control treated cultures (normalised against beta-actin) is shown below each protein of interest. **l** Western blotting analysis of PHOSPHO1, TNAP, nSMase2 in response a 24-h exposure PTH (50 nM) in MC3T3-C14 cells at different stages of differentiation. The fold change in fluorescence intensity against day 0 control treated cultures (normalised against beta-actin) is shown below each protein of interest. *N* = 3; *P < 0.05; **P < 0.01; ***P < 0.001 in comparison with controls

Initial experiments assessed the ability of varying concentrations of PTH to regulate *Phospho1*, *Alpl* and *Smpd3* expression by MC3T3-C14 cells. MC3T3-C14 cells were treated with 0.05–50 nM PTH for 24 h on day 6 of culture, when initial mineral formation could be observed with light microscopy. At concentrations of 0.5 nM and above, PTH significantly down-regulated *Phospho1* mRNA expression (*P* < 0.001; Fig. 2a). PTH increased *Alpl* expression at the two highest concentrations examined, 25 nM (*P* < 0.05) and 50 nM (*P* < 0.001), whereas it down-regulated *Smpd3* expression at all concentrations examined (*P* < 0.001; Fig. 2b, c). Western blotting confirmed that the changes in gene expression were replicated at the protein level (Fig. 2j).

We next examined the temporal effects of PTH on *Phospho1, Alpl and Smpd3* expression at day 7 of culture (matching the end point in experiments described in Fig. 2a–c). PTH rapidly down-regulated *Phospho1* after 15 min which was further exacerbated after 1-h and 6-h exposures (*P* < 0.001; Fig. 2d). By 24 h, the down-regulation of *Phospho1* by PTH was lessened (*P* < 0.05), and after 48-h and 72-h exposures *Phospho1* expression had returned to basal levels (Fig. 2d). Enhancement of *Alpl* expression by PTH was noted after 1 h (*P* < 0.05), peaking at 6 h (*P* < 0.001) before returning to control levels after 24-h and longer exposures (Fig. 2e). The expression pattern of *Smpd3* in response to PTH was similar to that of *Phospho1*; however, the speed of reduced expression and its normalisation was slower (Fig. 2f). Changes in protein levels of PHOSPHO1, TNAP and nSMase2 reflected their respective changes in gene expression (Fig. 2k). Notably, the expression levels of PHOSPHO1 and nSMase2 were less, and TNAP was greater after 24-h and 48-h PTH exposure (Fig. 2k). After a 72-h exposure all proteins were expressed at a comparable level to the control treated cultures.

Finally, the effect of PTH (50 nM, 24 h) on the expression of *Phospho1*, *Alp1* and *Smpd3* was assessed in MC3T3-C14 cells at different stages of differentiation. *Phospho1* and *Smpd3* regulation by PTH was not dependent on the differentiation status of the cell cultures, with both genes significantly down-regulated (*P* < 0.001) at 0, 7 and 10 days in culture (Fig. 2g, i). In contrast, PTH-induced expression of *Alpl* was more restricted and only reached significance at day 0 (*P* < 0.01; Fig. 2h). Little or no PHOSPHO1, nSMase2 or TNAP protein was expressed at day 0 in culture, and this was not altered by PTH treatment (Fig. 2l). At days 7 and 10, however, PTH decreased PHOSPHO1 and nSMase2 expression whilst increasing TNAP expression, consistent with the gene expression data (Fig. 2l).

### The Effects of Continuous PTH on Gene Expression in Murine Calvariae

**Fig. 3:**
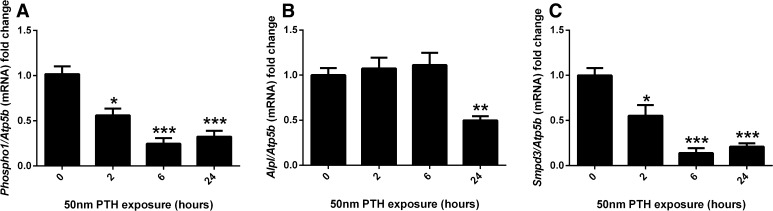
Regulation of key mineralisation genes by PTH in cultured calvariae. RT-qPCR analysis of **a** *Phospho1*, **b** *Alpl* and **c** *Smpd3* mRNA expression in response to various exposure times of PTH (50 nM). *N* > 3 per time point; *P < 0.05; ***P < 0.001 in comparison with control

Murine hemi-calvariae were treated with 50 nM PTH for 2, 6 and 24 h. A significant downregulation of both *Phospho1* and *Smpd3* expression in response to PTH was observed at 2 h (*P* < 0.05), 6 and 24 h (*P* < 0.001; Fig. 3a, c). Likewise, continuous PTH exposure induced a down-regulation of *Alpl* expression, albeit only with 24-h exposure (*P* < 0.01; Fig. 3b).

### The Effects of Cycloheximide on PTH on *Phospho1*, *Alpl* and *Smpd3* Gene Regulation

**Fig. 4:**
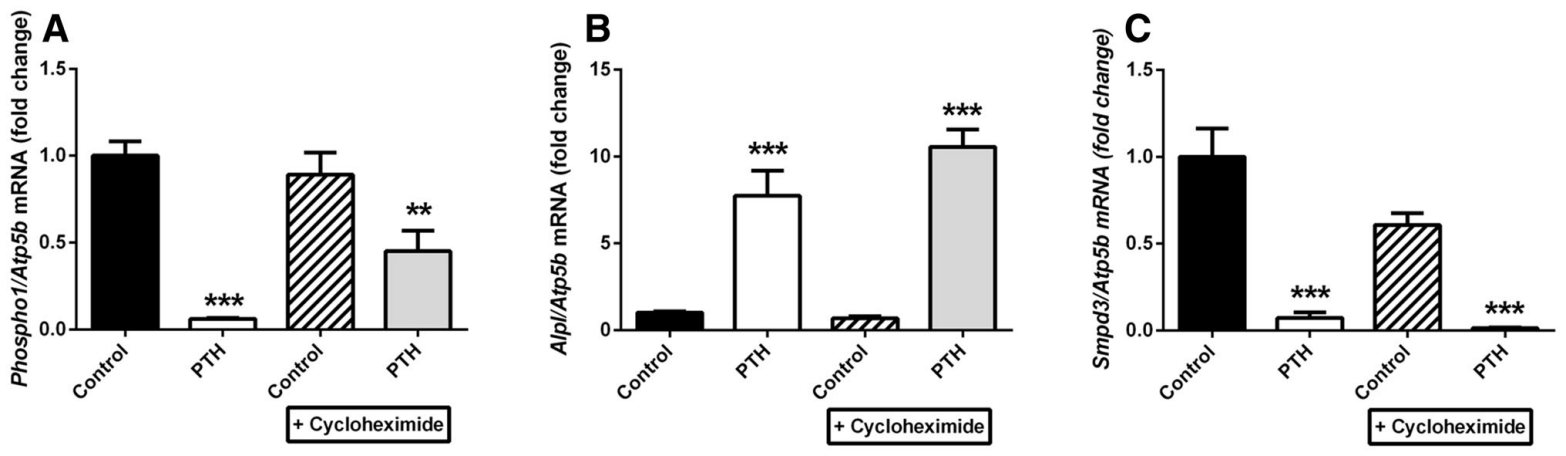
Effects of bPTH (1–34) on *Phospho1*, *Alpl* and *Smpd3* in MC3T3-C14 cells does not require protein synthesis. MC3T3-C14 cells were treated for 6 h with 50 nM PTH or vehicle control after a 2-h pre-treatment with 50 μM cycloheximide (CHX) or solvent control on day 7 in culture. **a** *Phospho1*, **b** *Alpl* and **c** *Smpd3* mRNA expression was assessed by RT-qPCR. *N* = 3; *P < 0.05; **P < 0.01; ***P < 0.001 in comparison with vehicle control

To determine whether PTH directly regulates *Phospho1*, *Alpl* and *Smpd3* gene expression, MC3T3-C14 cells were pre-treated for 1 h with the protein synthesis inhibitor cycloheximide (25 µM), followed by treatment with 50 nM PTH for 6 h. Cycloheximide treatment alone did not affect basal levels of *Phospho1*, *Alpl* or *Smpd3*. In the presence of cycloheximide, PTH reduced the expression of *Phospho1* (44.1 %, *P* < 0.01), but this was not to the same extent observed in the absence of cycloheximide (93.9 %, *P* < 0.001; Fig. 4a). Cycloheximide treatment did not affect the potent regulation of *Alpl* and *Smpd3* expression by PTH (Fig. 4b, c).

### Identification of the Intracellular Signalling Pathways Responsible for PTH Regulation of *Phospho1*, *Alpl* and *Smpd3* Expression

**Fig. 5:**
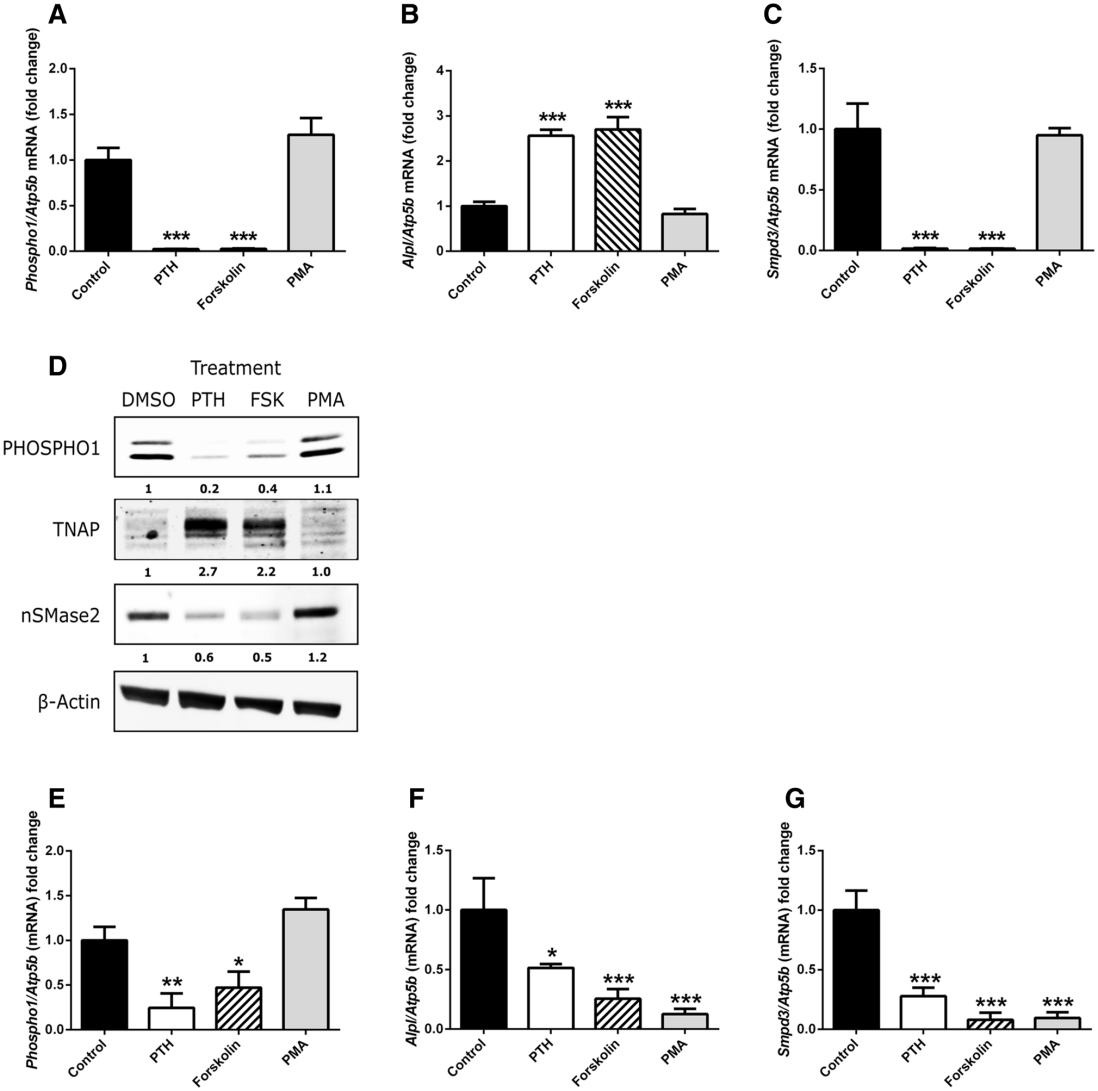
Investigation of signalling pathways regulating the effects of PTH. MC3T3-C14 cells were treated with 50 nM PTH, 25 µM forskolin, 100 nM PMA or vehicle control for 24 h on day 6 in culture. **a** *Phospho1*, **b** *Alpl* and **c** *Smpd3* mRNA expression was assessed by RT-qPCR. **d** Western Blotting analysis of MC3T3-C14 cells treated as described above. Hemi-calvariae were treated with 50 nM PTH, 25 µM forskolin, 100 nM PMA or vehicle control for 24 h. **e** *Phospho1*, **f** *Alpl* and **g** *Smpd3* mRNA expression was assessed by RT-qPCR. *N* = 3; *P < 0.05; **P < 0.01; ***P < 0.001 in comparison with vehicle control

**Fig. 6:**
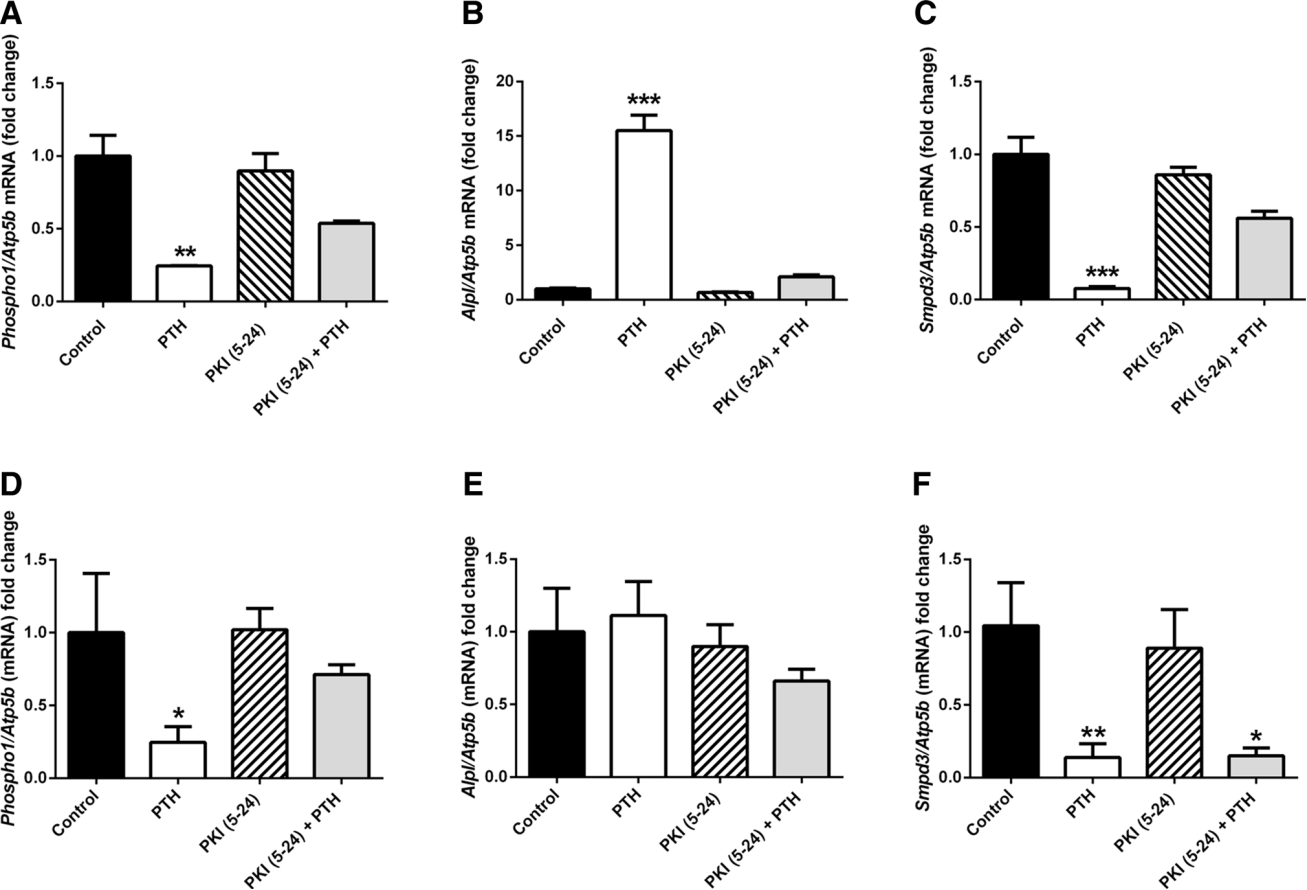
Inhibition of PKA signalling pathway abolishes the effects of PTH (1–34). MC3T3-C14 cells and hemi-calvariae were treated with 50 nM PTH or vehicle control for 6 h following a 1-h pre-treatment with 100 nM PKI (5–24) on day 6 of culture. **a** *Phospho1*, **b** *Alpl* and **c** *Smpd3* mRNA expression in MC3T3-C14 and **d** *Phospho1*, **e** *Alpl* and **f** *Smpd3* mRNA expression in hemi-calvariae, all assessed by RT-qPCR. *N* = 3; *P < 0.05; **P < 0.01, ***P < 0.001 in comparison with vehicle control

Binding of PTH to the G-protein-coupled receptor PTHR1 primarily activates cAMP/PKA and PKC signalling pathways [25]. To determine which of these pathways was utilised by PTH to regulate *Phospho1*, *Alpl* and *Smpd3* expression, we treated MC3T3-C14 cells and murine hemi-calvariae with the cAMP inducer forskolin (10 µM) or the PKC activator, PMA (100 nM) for 24 h. In MC3T3-C14 cells, forskolin replicated the effects of 50 nM PTH by inducing significant decreases in *Phospho1* (*P* < 0.001) and *Smpd3* expression (*P* < 0.001) whilst significantly increasing the expression of *Alpl* (*P* < 0.001) PMA treated cells displayed no differences in *Phospho1*, *Alpl* or *Smpd3* expression compared with control cultures (Fig. 5a–c). The gene expression results in MC3T3-C14 cells were confirmed at the protein level by western blotting (Fig. 5d). In hemi-calvariae, forskolin similarly replicated the effects of 50 nM PTH by inducing a significant decrease in *Phospho1* (*P* < 0.05) *Smpd3* (*P* < 0.001) *Alpl* (*P* < 0.001) expression (Fig. 5e–g). PMA-treated hemi-calvariae displayed no differences in *Phospho1* expression; however, the expression of *Alpl* and *Smpd3* was strongly down-regulated by exposure to PMA (*P* < 0.001). To confirm the role of the PKA signalling pathway as the mediator of the effects of PTH in MC3T3-C14 cells, cell cultures were treated with the PKA inhibitor PKI (5-24) (100 nM) alone or in the presence of PTH for 6 h. The addition of PKI (5-24) alone did not alter the basal expression of *Phospho1*, *Alpl* or *Smpd3*. The addition of the PKA inhibitor reduced the ability of 50 nM PTH to significantly inhibit the expression of *Phospho1* and *Smpd3* (Fig. 6a, c). Similarly, the induction of *Alpl* expression by PTH was abrogated by the co-incubation of PKI (5-24) (Fig. 6b). In cultured hemi-calvariae, PKI (5-24) inhibited the down-regulation of *Phospho1* by PTH, whereas the down-regulation of Smpd3 by PTH was not affected by PKI (5-24) (*P* < 0.001; Fig. 6f).

### Assessment of Transcription Factor Expression in Response to PTH in Cultured MC3T3 Cells and Murine Calvariae

**Fig. 7:**
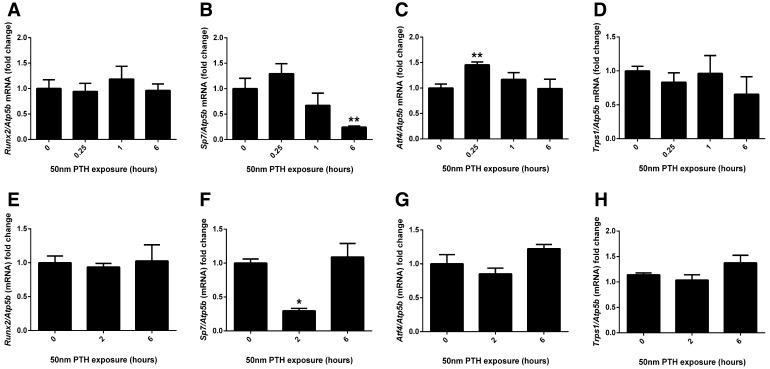
Transcription factor expression in response to short-term PTH exposure. **a** *Runx2,* **b** *Sp7,* **c** *Atf4* and **d** *Trps1* mRNA expression was assessed by RT-qPCR in MC3T3-C14 cell cultures in response to a short-term exposure to 50 nM PTH on day 7 of culture. **e** *Runx2,* **f** *Sp7,* **g** *Atf4* and **h** *Trps1* mRNA expression was assessed by RT-qPCR in cultured murine calvariae in response to a short-term exposure to 50 nM PTH. *N* = 3; *P < 0.05; **P < 0.01 in comparison with control-treated cultures

The expression of the transcription factors *Runx2, Sp7, Atf4 and Trps1* was assessed by RT-qPCR in MC3T3 cell cultures and cultured murine calvariae in response to a short-term PTH exposure. In MC3T3 cells, the expression of *Runx2* and *Trps1* did not change in response to PTH exposure (Fig. 7a, d). *Sp7* expression was reduced after a 6-h PTH exposure (*P* < 0.01; Fig. 7b), whereas Atf4 was enhanced after 15-min PTH exposure (*P* < 0.01; Fig. 7c). In cultured murine calvariae, the expression of *Runx2*, *Atf4* and *Trps1* was not altered in response to PTH exposure (Fig. 7e, g, h). *Sp7* expression was significantly down-regulated after a 2-h PTH exposure (*P* < 0.05; Fig. 7f).

### Investigating the Combined Effects of Continuous PTH and BMP-2 Exposure on Gene Expression in MC3T3 Cells

**Fig. 8:**
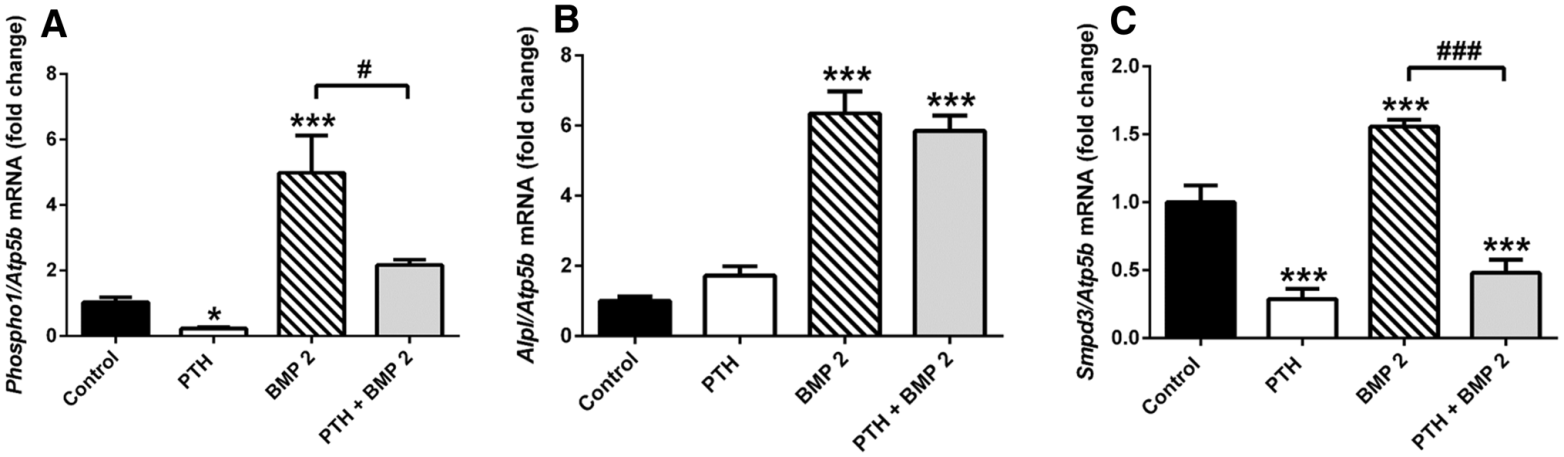
Combined effects of continuous PTH and BMP-2 exposure on *Phospho1*, *Alpl* and *Smpd3* expression in MC3T3-C14 cells. **a** *Phospho1,* **b** *Alpl and* **c** *Smpd3* mRNA expression was assessed by RT-qPCR in MC3T3-C14 cell cultures in response to a 24 h exposure to 50 nM PTH, 300 ng/mL BMP-2 or both on day 6 of culture. *N* = 3; *P < 0.05; ***P < 0.001 in comparison to control treated cultures; ^**###**^ *P* < 0.001 in comparison with BMP-2-treated cultures

To investigate the effects of continuous PTH exposure in MC3T3 cells treated with BMP-2, MC3T3 cell cultures were cultured with either 50 nM PTH, 300 ng/mL BMP-2 or both for 24 h on day 6 of culture, and the expression of *Phospho1*, *Alpl* and *Smpd3* was assessed by RT-qPCR. BMP-2 exposure significantly induced the expression of *Phospho1*, *Alpl* and *Smpd3* (*P* < 0.001; Fig. 8a–c). The stimulatory effect of BMP-2 on *Phospho1* expression was nullified by the dual exposure of PTH which caused *Phospho1* expression to return to unstimulated control levels (Fig. 8a). In contrast, the *Smpd3* expression in cells treated with PTH or PTH and BMP-2 was similar, suggesting that *Smpd3* expression was highly sensitive to PTH exposure (*P* < 0.001; Fig. 8c). The increased expression of *Alpl* by BMP-2 was not affected by the co-incubation of PTH.

## Discussion

The role for TNAP in promoting ECM mineralisation has long been recognised and more recent studies have also identified PHOSPHO1 and nSMase2 as important regulators of the biomineralisation process [7, 11, 26]. It is likely that nSMase2 and PHOSPHO1 function together to control the initiation of mineralisation via the liberation of Pi within the sheltered confines of osteoblast and chondrocyte MVs [1]. This possibility is supported by the observation that both PHOSPHO1 and nSMase2 are present in MV’s [27]. In contrast, TNAP is bound, by means of a glycosylphosphatidylinositol anchor, to the outer leaflet of MV membranes, whereby its pyrophosphatase activity modulates the extravesicular Pi/PPi ratio so as to provide an environment conducive to mineral propagation [1, 11, 13]. The complete ablation of ECM mineralisation noted in *Phospho1* ^−/−^ *;Alpl* ^−/−^ double-knockout mice underscores the importance and the non-redundant functions of these two phosphatases in regulating skeletal mineralisation [11]. Recent studies have further shown that MV’s initiate mineralisation by a dual mechanism: PHOSPHO1-mediated intravesicular generation of Pi and phosphate transporter-mediated influx of Pi [28]. Despite knowledge of the function of these mineralisation-dependant proteins, there exists a hiatus in the knowledge surrounding their regulation. However, recent transcriptome sequencing analysis revealed that *Phospho1* is regulated by PTH in osteocytes [16]. This study was the impetus for this present investigation, where we sought to determine whether the catabolic effects of continuous PTH may be attributed to the regulation of PHOSPHO1 expression by osteoblasts, and also how this regulation may be coordinated with TNAP and nSMase2 expression.

This study has revealed that PHOSPHO1, nSMase2 and TNAP display coordinated and significant increases in the levels of transcript and protein prior to the onset of ECM mineralisation in the MC3T3-C14 osteoblast-like cell line. In particular, the similarities in the temporal rise of PHOSPHO1 and nSMase2 protein bolster the idea that these phosphatases work in tandem to generate Pi within MV during the initiation of mineralisation. Furthermore, PHOSPHO1 has recently been implicated in the biogenesis of MV [28], and as such its expression prior to the onset of mineralisation is crucial for the formation of MVs that ultimately contain a precipitated carbonate-substituted hydroxyapatitic mineral phase [29, 30]. The higher expression of TNAP late on (day 10) noted in the time course of this study, highlights its role in the later stages of mineralisation where it allows the propagation of hydroxyapatite within the ECM beyond the confines of the MV membrane [1, 10].

This study provides the first evidence for the potent regulation of *Phospho1* and *Smpd3* by PTH in osteoblast-like cells and cultured calvariae. Our data build on previous investigations which have sought to uncover the regulation of mineralisation-dependent genes by growth factors and hormones. The enhancement of nSMase2 expression by BMP-2 signalling was recently reported in chondrocytes and C2C12 myoblast cells [31, 32]. In this present study we have confirmed and expanded these findings, by revealing that continuous PTH exposure is able to significantly down-regulate *Smpd3* expression even in the presence of BMP-2. *Phospho1* expression was similarly enhanced by BMP-2 exposure, with concomitant PTH exposure restoring expression levels to those of control cultures. The effects of BMP signalling on PHOSPHO1 regulation and the interaction between BMP and PTH signalling are intriguing and worthy of further investigation.

Whilst we found no interaction between PTH and BMP-2 in the regulation of *Alpl* expression, the effects of PTH alone on *Alpl* expression have been well documented. For example, intermittent exposure to PTH has been shown to stimulate ALP activity and bone nodule formation [33]. However, it should be noted that the inhibition of osteoblast differentiation by PTH has been reported in other studies [34, 35], and this variation in response may be explained by differences in osteoblast differentiation stage and PTH exposure time and dosage. PTH can exert entirely opposing effects on bone which depend upon the length of exposure. Intermittent exposure to PTH is anabolic to the skeleton, with reactivation of bone lining cells [36], inhibition of osteoblast apoptosis [37] and down-regulation of sclerostin, the potent negative regulator of Wnt signalling and bone formation [38]. Currently, rhPTH (1-34) (Teriparatide) is the only anabolic therapy for osteoporosis aimed at preventing both vertebral and non-vertebral fractures in post-menopausal women [39]. Contrastingly, continuous exposure to PTH, as observed in hyperparathyroidism, is catabolic to bone, increasing osteoclastogenesis through upregulation of RANKL and inhibition of osteoprotegerin expression [22]. Knowledge of the effects of continuous exposure to PTH is, however, limited with regard to the regulation of mineralisation-promoting enzymes despite an overall increase in the remodelling rate observed in hyperparathyroidism [21]. The nature of the PTH administration described in this study, whereby PTH is added to the culture medium, and the medium is not changed before the experiments are stopped for collection of RNA/protein, which most closely resembles a continuous exposure to PTH. Indeed a number of investigations to date have demonstrated this method of PTH exposure to better model the catabolic effects of PTH [40, 41]. With bovine PTH (1-34) having a reported half-life of 10–12 h in vitro a biologically effective dose of ~3 nM should have remained in our cultures after 48 h. However, as our time exposure studies show (Fig. 2d–e), *Phospho1* and *Alpl* gene expression levels were normalised by 48 h. This suggests the lack of a biologically effective dose of PTH at this time point. In contrast, *Smpd3* expression remained significantly down-regulated at 48 h possibly reflecting the increased sensitivity of this gene to PTH challenge (Fig. 2f). These data bring into question the levels of biologically active PTH remaining in our experimental cultures and highlight the limitations of this approach. However, this method of PTH exposure is in contrast to in vitro studies which seek to model the effects of intermittent PTH, whereby PTH is added to cell cultures for 1–6 h per 24-h or 48-h incubation cycle [40, 42]. The findings presented here show for the first time that continuous administration of PTH to osteoblast cultures inhibits the expression of *Phospho1* and *Smpd3.*

The reduction of PHOSPHO1 and nSMase2 may provide a novel mechanism by which continuous PTH exposure results in decreased bone mineral density. Indeed, continuous exposure to PTH reduced the extent of matrix mineralisation in MC3T3-C14 cell cultures (Fig. 1g) corroborating existing reports which assessed this protocol in rat calvarial osteoblast cultures [40]. Recent studies have provided data which highlight a role for PHOSPHO1 and nSMase2 in the biogenesis of matrix vesicles [28, 43, 44]. It is possible, therefore, that the reduced mineralisation of MC3T3-C14 cell cultures observed in response to continuous exposure to PTH is due to both a decreased number of MVs being released and a decrease in their ability to initiate amorphous hydroxyapatite formation. Despite this, *Alpl* expression was induced in response to PTH in MC3T3-C14 cell cultures. An enhancement of Alpl expression and TNAP activity in response to a continuous exposure to PTH has previously been shown in periodontal ligament cells [45] and in the UMR-106 cell line [46], respectively. Even with this body of evidence in mind, the differential regulation of *Phospho1* and *Alpl* by PTH is perhaps surprising. The genetic ablation of *Phospho1* in the mouse leads to reduced serum TNAP activity and reduced *Alpl* expression in cultured chondrocytes derived from these mice [11]. Importantly, cultured hemi-calvariae exposed to continuous PTH exposure for 24 h displayed a down-regulation of *Alpl* expression (Fig. 3b). The ex vivo culture of calvariae may provide a more physiologically relevant model of osteoblast behaviour due to the maintenance of both cell–cell and cell–matrix interactions in three-dimensional space.

**Fig. 9:**
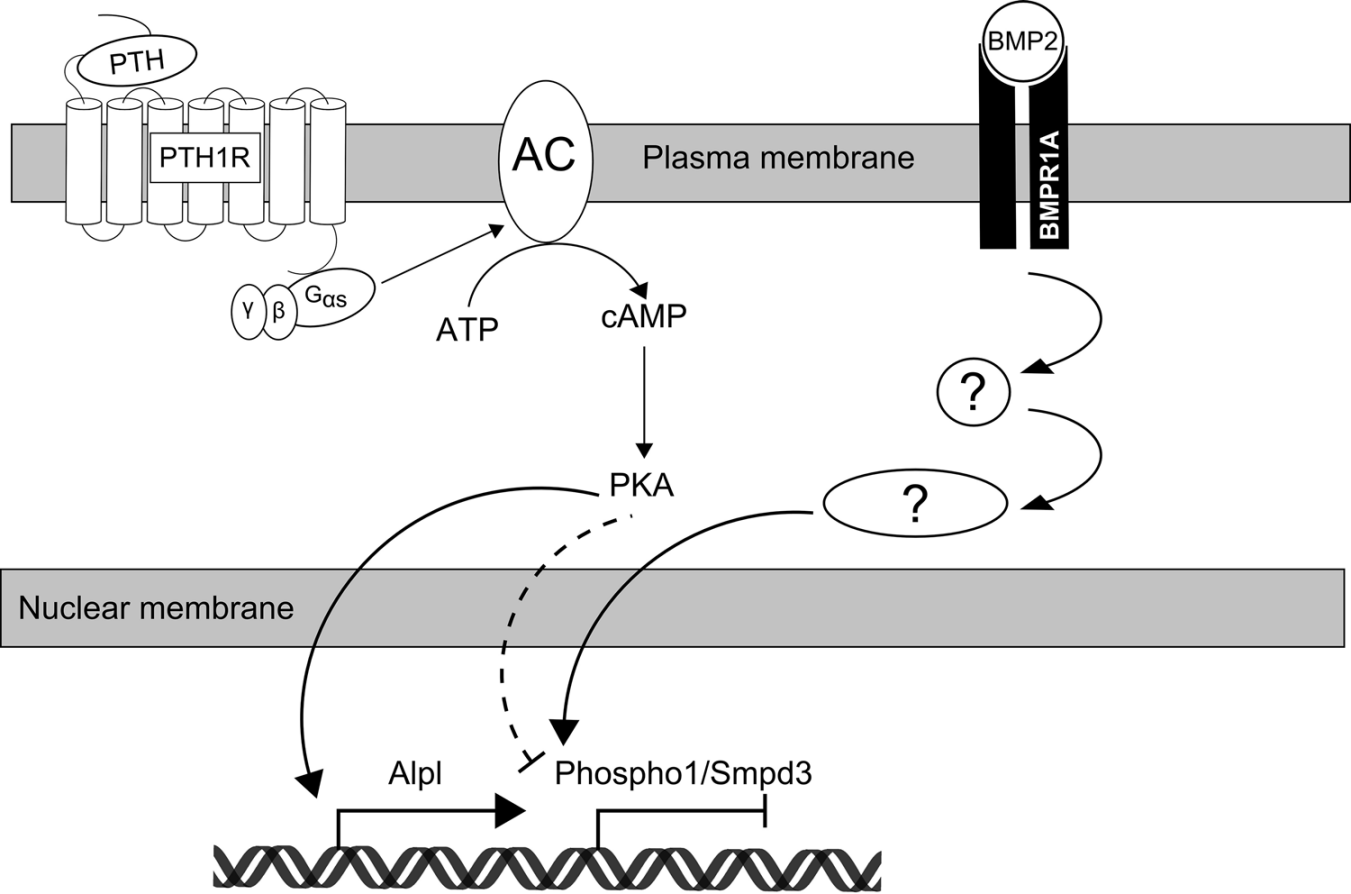
Schematic representation of the signalling pathways mediating PTH and BMP-2 induced regulation of *Phospho1*, *Smpd3* and *Alpl* in osteoblast cells. Recapitulation of the effects of PTH, through the use of the cAMP agonist, forskolin, and inhibition of the effects of PTH through the use of the PKA inhibitor, PKI (5–24) have implicated the cAMP/PKA pathway in mediating the down-regulation of *Phospho1* and *Smpd3*, and the enhancement of *Alpl* mRNA expression in response to PTH treatment. Initial studies have identified BMP-2 as an inducer of *Phospho1*, *Smpd3* and *Alpl* expression, although the intracellular signalling pathway is yet to be elucidated. PTH, parathyroid hormone; PTH1R, parathyroid hormone receptor 1; AC, adenylyl cyclase; ATP, adenosine triphosphate; cAMP, cyclic adenosine monophosphate; PKA, protein kinase A; BMP2, bone morphogenic protein 2; BMPR1A, bone morphogenic protein receptor 1A

To enact its effects, PTH binds to the PTH 1 receptor (PTH1R), a G-protein-coupled receptor, through a receptor binding domain between amino acids 15–24 [47]. Both PKA and PKC signal transduction pathways may be activated by binding of PTH to PTH1R. The effects of PTH are primarily elicited through the PKA pathway whereby, stimulatory Gαs proteins activate adenylate cyclase, with subsequent production of cAMP and phosphorylation of PKA [17]. A limited number of genes, for example those encoding the insulin-like growth-factor-binding protein 5 and transforming growth factor β1, have been found to be regulated by Gαq activation, which results in PKC activation, 1,4,5-inositol triphosphate production and a rise in intracellular Ca2^+^ [25]. A critical role for the cAMP-PKA pathway in mediating the effects of PTH on PHOSPHO1, nSMase2 and TNAP expression in osteoblast cell cultures model has been revealed in this study. Firstly, the adenylyl cyclase agonist, forskolin, replicated the effects of PTH, whereas exposure to the PKC agonist, PMA, caused no effects. Furthermore, specific inhibition of the cAMP activation of PKA by the synthetic peptide PKI (5-24) abrogated the effects of PTH on *Phospho1*, *Alpl* and *Smpd3* expression (Fig. 9). In support of a role for PKA signalling in the downregulation of the mineralisation-dependent enzymes, PHOSPHO1 and nSMase2, studies to date have shown the inhibition of both MC3T3-E1 matrix mineralisation and bone nodule formation by cultured rat calvarial cells with long-term exposure to forskolin [48, 49]. Our studies involving cycloheximide reveal that this genetic regulation is not dependent on protein synthesis. It is noted that cycloheximide appeared to partially affect the downregulation of *Phospho1* by PTH exposure (inducing a 49.5 % decrease as opposed to a 93.9 % decrease by PTH alone). Despite this, it is well accepted that cycloheximide can exert off-target effects [50], and these may account for this difference. Finally, we assessed the expression of the osteogenic transcription factors *Runx2*, *Sp7* and *Atf4* and *Trps1*, which has recently been implicated in the regulation of *Phospho1* in a preodontoblastic cell line [51]. In both cultured murine calvariae and MC3T3-C14 cells, *Sp7* expression was reduced in response to PTH. This suggests that osterix (encoded by the *Sp7* gene), one of the master transcription factors regulating osteogenic differentiation, may be an important transcription factor in the regulation of both *Phospho1* and/or *Smpd3* expression. The study of the transcriptional and epigenetic regulation of these mineralisation-dependent genes requires further study, and it is of interest to note that Runx2 overexpression in mouse limb bud cells has been shown to result in elevated expression of both *Phospho1* and *Smpd3* [52].

These data are novel for the PTH control of *Phospho1* and *Smpd3* expression, but previous reports have demonstrated the involvement of the cAMP-PKA pathway in the regulation (both positive and negative) of TNAP activity in a variety of cell culture models of mineralisation [33, 40, 45, 46]. The PTH-dependent enhancement of TNAP activity has been shown to be enacted through cAMP-PKA-mediated activation of p38 MAP kinase [53]. In the cultured hemi-cavariae experiments, cAMP stimulation by forskolin simulated the effects of PTH by inducing a downregulation of *Phospho1, Alpl and Smpd3*. In contrast to MC3T3-C14 cell cultures, however, PMA similarly induced the down-regulation of *Alpl* and *Smpd3*, and blockade of PKA activation by PKI (5-24) did not affect the PTH-induced downregulation of *Smpd3.* To date, *Phospho1* has been shown to be exclusively expressed by osteoblasts, hypertrophic chondrocytes, odontoblasts and calcifying vascular smooth muscle cells [8, 9, 54]. *Smpd3* and *Alpl*, on the other hand, are more broadly expressed amongst different tissues and cell types. Although providing a more physiological environment, the presence of osteocytes, osteoclasts and stromal cells, as well as osteoblasts at differing stages of development in the ex vivo calvariae model, may be confounding our investigation of the signalling pathways in this model.

In summary, the data presented here provide evidence for of a temporal rise in the expression of key mineralisation effector enzymes, PHOSPHO1, TNAP and nSMase2, prior to the onset of in vitro mineralisation. This finding highlights the importance of these enzymes in this process. Furthermore, the potent negative regulation of *Phospho1* and *Smpd3* by continuous exposure to PTH may provide an additional means of explaining the poor bone quality observed in response to sustained and elevated exposures to PTH.

## Materials and Methods

### MC3T3-Clone 14 Cell Culture

MC3T3 subclone 14 cells (MC3T3-C14) (ATCC, CRL-2594™) previously characterised as a highly mineralising clone of the MC3T3-E1 cell line [13, 55] were plated at 1 × 104 cells/cm2 in six-well plates and cultured in maintenance medium (α-MEM containing 10 % FBS and 0.5 % Gentamicin) (Invitrogen, Paisley, UK) in a humidified atmosphere (37 °C, 5 % CO2). Upon confluency, maintenance media was replaced with mineralisation media (maintenance medium supplemented with 50 µg/ml L-ascorbic acid and 1.5 mM calcium chloride) (Sigma-Aldrich, Dorset, UK). Supplementation with calcium chloride successfully induces ECM mineralisation and negates the need for exogenous phosphate sources (e.g. beta-glycerophosphate) and therefore potential ectopic mineralisation or enhanced induction of *Alpl* expression [13, 56, 57]. Media was changed every 2–3 days for up to 10 days.

To examine the effects of continuous exposure of PTH on MC3T3-C14 gene and protein expression, the media was replaced with fresh mineralisation media containing either bPTH (1-34) (Sigma-Aldrich) (referred to as PTH) at various concentrations (0.5–50 nM) or vehicle (PBS) and for various lengths of time (15 min–72 h). Unless otherwise stated, cells were cultured for 6 days (from confluency) under osteogenic conditions prior to the addition of PTH in order to avoid suppression of osteoblast differentiation [58, 59]. In experiments utilising BMP-2, cell cultures were treated with recombinant human BMP-2 (300 ng/mL; R & D Systems, Abingdon, UK) for 24 h. To investigate the signalling pathways mediating the effects of PTH, cells were also treated with forskolin (25 µM; Sigma-Aldrich), phorbol 12-myristate 13-acetate (PMA, 100 nM; Sigma-Aldrich), and PKI (5-24) (100 nM; Santa Cruz Biotechnology, Texas, USA) or appropriate vehicle controls for the time indicated. To assess the requirement of protein synthesis in the PTH regulation of *Phospho1*, *Alpl* and *Smpd3*, cells were pre-incubated for 2 h with cycloheximide (25 µM; Sigma-Aldrich) or DMSO control prior to stimulation with either 50 nM PTH or PBS for 6 h.

### Murine Calvariae Culture

Calvariae from 4- to 5-day-old C57Bl/6 J pups were dissected under sterile conditions. Hemi-calvariae were formed by cutting along the sagittal suture of the calvaria. Hemi-calvariae were cultured in 24-well plates in 350 µL of calvaria culture medium (α-MEM containing 0.2 % w/v bovine serum albumin, 0.05 mg/ml gentamicin, 1.25 µg/mL Amphotericin B and 5 µg/ml L-ascorbic acid). After 24 h hemi-calvariae were treated with 50 nM PTH, forskolin (25 µM), PMA (100 nM) and PKI (5-24) (100 nM) for the times indicated.

### Quantification of Extracellular Matrix Mineralisation

The effect of continuous PTH exposure on the ECM mineralisation of MC3T3-C14 cells was assessed by alizarin red staining and quantification. To examine the effects of continuous PTH on the ECM mineralisation, cell cultures were exposed to either 50 nM PTH or PBS control for 10 days under osteogenic conditions; media changes, including fresh PTH or control, were performed every 48 h. On day 10 of culture, cells cultures were fixed in 4 % paraformaldehyde for 5 min at room temperature. Cell monolayers were stained with aqueous 2 % (w/v) Alizarin red solution (Sigma-Aldrich) for 5 min at room temperature. The bound stain was solubilised in 10 % cetylpyridinium chloride (Sigma-Aldrich) and the optical density of the resultant eluted solution measured by spectrophotometry at 570 nm.

### Analysis of Gene Expression by Quantitative Real-Time PCR

MC3T3-C14 cultures and hemi-calvariae were stopped at the desired time in culture and total RNA was extracted using the RNeasy mini kit and RNeasy mini lipid kit, respectively, (Qiagen, Crawley, UK) according the manufacturer’s instructions. RNA concentration was assessed by absorbance at 260 nm and purity by A260/280 ratio using a NanoDrop spectrophotometer (Fisher Scientific, Loughborough, UK). Reverse transcription of RNA was carried out using Superscript II (Invitrogen) according to the manufacturer’s instructions. Gene expression analysis, using the SYBR green detection method was performed on a Stratagene Mx3000P real-time qPCR system (Stratagene, California, USA). Samples were assessed in triplicate and normalised against the house keeping gene, *Atp5b* (Primer Design, Southampton, UK) as previously done in similar studies [13]. Relative gene expression was calculated using the ΔΔCt method [60] and expressed as a fold change compared to control. The primers used to amplify *Phospho1*, *Smpd3,* *Alpl, Runx2, Sp7, Atf4* and *Trps1* are shown in Table 1.

**Table 1:** Primer sequences used to examine gene expression by RT-qPCR

*Phospho1*	Forward primer	5′-TTCTCATTTCGGATGCCA-3′	PrimerDesign, Southampton, U.K.
	Reverse primer	5′-TGAGGATGCGGCGGAAT-3′	
*Alpl*	Forward primer	5′-CTGCCACTGCCTACTTGTGT-3′	MWG Eurofins, Munich, Germany
	Reverse primer	5′-GATGGATGTGACCTCATTGC-3′	
*Smpd3*	Forward primer	5′-ACACGACCCCCTTTCCTAATA-3′	MWG Eurofins, Munich, Germany
	Reverse primer	5′-GGCGCTTCTCATAGGTGGTG-3′	
*Runx2*	Forward primer	5′-TGGCCGGGAATGATGAGAAC-3′	MWG Eurofins, Munich, Germany
	Reverse primer	5′-TGAAACTCTTGCCTCGTCCG-3′	
*Sp7*	Forward primer	Not disclosed by supplier	Qiagen, Crawley, U.K.
	Reverse primer	Not disclosed by supplier	
*Atf4*	Forward primer	5′-GTGGCCAAGCACTTGAAACC-3′	MWG Eurofins, Munich, Germany
	Reverse primer	5′-GGAAAAGGCATCCTCCTTGC-3′	
*Trps1*	Forward primer	5′-ACAACGGCGAGCAGATTATTAG-3′	MWG Eurofins, Munich, Germany
	Reverse primer	5′-TAGTCAATGAACCCTGGGCTTCGTA-3′	

### Western Blotting

Protein was extracted from cell monolayers using RIPA buffer (Invitrogen) with complete protease inhibitors (Roche, East-Sussex, UK). The concentration of the resultant protein lysates was determined by DC assay (Bio-Rad, Hemel Hempstead, UK). Proteins (10 µg) were separated on a 10 % Bis–Tris gel and transferred to a nitrocellulose membrane. Membranes were blocked with Odyssey blocking buffer (LI-COR Biosciences, Nebraska, USA) for 1 h at room temperature and probed overnight at 4 °C with anti-PHOSPHO1 (AbD Serotec, Martinsreid/Planegg, Germany), anti-TNAP (R&D, Abingdon, UK), anti-nSMase2 (Santa Cruz Biotechnology) and anti-β-actin (Cell Signalling Technology, Hitchin, UK) antibodies diluted 1:1000, 1:500, 1:500 and 1:1000 respectively in Odyssey blocking buffer with 1.875 % Tween 20. After washing in PBS, membranes were probed with the appropriate secondary antibodies for 50 min at room temperature (1:1250 dilution in Odyssey blocking buffer). The resulting blots were subsequently washed in PBS and visualised using the LI-COR Odyssey infrared scanner and software (LI-COR biosciences) with a scan resolution of 169 µm. Quantification of the fluorescence of each protein of interest was performed using Image Studio Lite version 5.0 (LI-COR biosciences) and normalised against beta-actin. The resulting quantification was expressed as fold change compared with control-treated samples.

### Statistical Analysis

Data analysis was performed using GraphPad Prism 6 (GraphPad Prism Inc., California, USA), and data are presented as mean ± S.E.M. Comparisons between datasets were carried out using ANOVA or two-way ANOVA with post hoc Tukey’s tests where appropriate. *P* < 0.05 was considered to be significant and noted as ‘*’; *P* values of < 0.01 and < 0.001 were noted as ‘**’ and ‘***,’ respectively.
